# Infection as an indicator and additional factor for consideration of ECMO circuit change-out: A call for further research

**DOI:** 10.1051/ject/2024034

**Published:** 2025-03-07

**Authors:** Salman Pervaiz Butt, Stephanie Thomas, Salman Abdulaziz

**Affiliations:** 1 Interim Manager Perfusion Services, Heart Vascular and Thoracic Institute, Cleveland Clinic Abu Dhabi PO BOX 112412 United Arab Emirates; 2 Consultant Medical Microbiologist, Manchester University Foundation Trust Manchester M13 9WL UK; 3 Consultant of Cardiovascular Critical Care, Co-Chair of ECMO Task Force, Department of Health PO Box 5674 20224 United Arab Emirates

Dear Editor

We write in response to our recently published article, entitled *“Improving ECMO therapy: Monitoring oxygenator functionality and identifying key indicators, factors, and considerations for changeout”* [1]. While we continue to support our conclusion, that thrombosis within the membrane oxygenator is a significant contributor to dysfunction, and that the decision for circuit change-out requires considering factors such as fibrinogen levels, blood gases, plasma-free haemoglobin, D-dimers, platelet function, flows, pressures, and anticoagulation strategy, we feel that we overlooked the influence of infection on coagulation and its role as a possible additional factor that might also determine changeout of the system.

We now also propose, that there may be a significant relationship between infection and circuit dysfunction which has not previously been explored or fully described [2]. It is widely reported in the literature that the inflammatory state associated with COVID-19 infection may amplify the proinflammatory effect of ECMO [3]. The clinical consequences of this are bleeding and thrombosis, which may negatively impact circuit function necessitating changeout.

However, we suggest that in addition to viral infection, bacterial and fungal infection, particularly in those patients with positive blood cultures where pathogenic organisms are circulating in the bloodstream, around the circuit, and through the oxygenator, may provoke inflammatory responses and contribute to clot formation and build up within the oxygenator, potentially compromising oxygenator functionality.

The mechanisms by which infection-associated thrombosis is induced, maintained, and resolved are poorly understood, as is the contribution thrombosis makes to host control of infection and pathogen spread. The key difference between infection-associated thrombosis and thrombosis in other circumstances is a stronger inflammation-mediated component caused by the presence of the pathogen and its products. This inflammation triggers the activation of platelets, which may accompany damage to the endothelium, resulting in fibrin deposition and thrombus formation. This process is often referred to as thrombo-inflammation [4]. Therefore, we hypothesis that bacterial and fungal bloodstream infection may also be a factor to consider when contemplating oxygenator changeout.

In addition and conversely, we also suggest that clots residing within the oxygenator due to other thrombotic mechanisms as described in the author’s paper [1], may offer favourable conditions for microbial adherence and biofilm formation, increasing the risk of microbial persistence in the circuit [5]. This would be of particular significance for those pathogens commonly associated with infection in the Intensive Care patient, such as coagulase-negative staphylococcus, *Pseudomonas aeruginosa* and Candida species. These pathogens form biofilm readily on biotic or abiotic surfaces, which allows evasion from the host immune system and promotes antibiotic resistance.

We suggest a two-way relationship between infection and clot formation in the ECMO circuit, highlighting the interconnected nature of mechanical and infectious complications in critically ill patients requiring extracorporeal support. We suggest that further research is warranted to understand the complex relationship between bacterial and fungal bloodstream infection, time to clot formation and circuit dysfunction. This should be preceded by an extensive literature review on the subject, followed by recommendations for further, targeted research. Studying such a complex multifactorial system is all the more challenging in the ECMO population which typically consists of patients with profound heterogeneity of characteristics. A better understanding may allow the instigation of early pre-emptive measures, including bloodstream surveillance, adjustment of blood thinners, targeted antibiotics with antibiofilm activity and untimely guidance on the optimisation of oxygenator changeout, thereby optimizing ECMO therapy.

We humbly submit this proposal for consideration by your journal’s editorial board.
